# Experiences of discrimination across the transition to parenthood and postpartum depression severity among Black women

**DOI:** 10.1017/S003329172510113X

**Published:** 2025-07-17

**Authors:** Alison Hipwell, Kelsey Magee, Kate Keenan, Irene Tung, Ashley Hill, Ashley Stiller, Allysa Quick, Michele Levine, Sierra Strickland, Melanie Custodio

**Affiliations:** 1 Department of Psychiatry, University of Pittsburgh, Pittsburgh, PA, USA; 2 Department of Psychology, University of Pittsburgh, Pittsburgh, PA, USA; 3 Department of Psychiatry and Behavioral Neuroscience, University of Chicago, Chicago, IL, USA; 4 Department of Psychology, California State University, Dominguez Hills, Carson, CA, USA; 5 Division of Community Health Science, School of Public Health, University of Illinois Chicago, Chicago, IL, USA

**Keywords:** discrimination, longitudinal, pregnancy, pre-pregnancy, postpartum depression

## Abstract

**Background:**

Postpartum depression is prevalent among Black women and associated with intersecting systemic factors and interpersonal discrimination. However, gaps remain in understanding pregnancy-related changes in discrimination experiences that influence postpartum mental health and could inform preventive interventions. We hypothesized that young Black women would experience increasing levels of discrimination across the transition to parenthood, heightening depression risk relative to non-pregnant peers.

**Methods:**

Participants comprised 335 Black primiparous women (ages 17-30 at delivery) and 335 age- and discriminationmatched non-pregnant controls from the Pittsburgh Girls Study. Self-reported discrimination experiences were collected at four timepoints: two years pre-pregnancy, one year pre-pregnancy, pregnancy, and one year postpartum for the childbearing sample, with corresponding data from the non-pregnant sample across the same interval (matched pairwise).

**Results:**

Linear increases in discrimination were observed for the nonpregnant participants (*B_S_* = .480, SE = .090, *p* <.001), while childbearing participants showed no overall changes, though younger age predicted greater increases over time. For childbearing participants, both baseline discrimination (*B_I_* = .626, *SE* = .077, *p* < .001) and increasing discrimination (*B_S_* = 2.55, *SE* = .939, *p* < .01) predicted postpartum depressive symptoms, controlling for pre-pregnancy depression. Among non-pregnant participants, only baseline discrimination predicted later depression (*B_I_* = .912, *SE* = .081, *p* < .001).

**Conclusions:**

Experiencing increasing levels of interpersonal discrimination across the transition to parenthood may heighten postpartum depression risk among young Black women, indicating a need for interventions supporting well-being and promoting resilience before, during and after pregnancy.

## Introduction

Postpartum depression, affecting 9%–37% of new mothers (Bauman, 2020; Gavin et al., 2005; Norhayati, Hazlina, Asrenee, & Emilin, 2015), is a major public health concern that disproportionately impacts women of color in the United States (Docherty, Stoyles, Najjar, & Woolley, 2022; Liu, Giallo, Doan, Seidman, & Tronick, 2016; Van Niel & Payne, 2020). Racism and race-related stressors, operating on interpersonal and structural levels, are major drivers of mental health disparities (Clark, Anderson, Clark, & Williams, 1999; Geronimus, Hicken, Keene, & Bound, 2006; Pascoe & Smart Richman, 2009), which likely extend to the peripartum period (Segre, Mehner, & Brock, 2021). This disparity is evident in surveillance data showing that being upset by racial discrimination in the 12 months prior to childbirth was associated with a two- to three-fold increase in postpartum depressed mood after accounting for covariates such as stressful life events (Segre et al., 2021; Weeks, Zapata, Rohan, & Green, 2022). Furthermore, among Black women, experiences of interpersonal discrimination (e.g. being hassled, disrespected, receiving inferior treatment) are associated with more severe depressive symptoms when assessed during pregnancy (Bennett et al., 2010; Bower, Geller, Jeffers, McDonald, & Alhusen, 2023; Canady, Bullen, Holzman, Broman, & Tian, 2008) or in the postpartum period (Heldreth et al., 2016).

Most peripartum research to date has considered discrimination experiences assessed retrospectively or at a single timepoint concurrent with measures of mental health. The dearth of prospective, longitudinal studies is concerning given that research with non-childbearing Black individuals shows that experiences of discrimination tend to (1) increase with age and across emerging adulthood (Greene, Way, & Pahl, 2006; Seaton, Caldwell, Sellers, & Jackson, 2008) and (2) precede increases in depressive symptoms (Brown et al., 2000; Schulz et al., 2006). Developmental studies have also shown that increasing, rather than stable or constant, trajectories of discrimination are associated with greater risk for subsequent depression (Bécares & Zhang, 2018; Brody et al., 2006; White, Bell, Huang, & Williams, 2020). Such results highlight the importance of modeling dynamic change in exposure to discrimination and examining the influence of these experiences during well-defined developmental periods (Gee, Walsemann, & Brondolo, 2012).

Black feminist and intersectionality theories highlight pregnancy as a critical life stage when Black women may face increased levels of discrimination due to negative societal stereotypes concerning sexuality and Black womanhood (Collins, 2000; Crenshaw, 1989; Essed, 1991). Indeed, studies have shown that Black women experience pregnancy-related stress resulting from assumptions regarding marital status, parity, and reliance on public assistance in both public (Mehra et al., 2020; Rosenthal & Lobel, 2020) and obstetric settings (McLemore et al., 2018; Slaughter-Acey et al., 2019). One noteworthy longitudinal study examined changes in experiences of discrimination at four time points (second and third trimesters of pregnancy and at 6 and 12 months postpartum) in a racially diverse sample (33% Black) of young women (14–21 years) recruited from community hospitals and health centers (Rosenthal et al., 2015). Lagged analyses showed that increases in discrimination predicted increases in depressive symptoms at subsequent time points for the overall sample, but patterns of change varied across different ages. Specifically, 14- and 15-year olds reported increases in discrimination through 6 months and then a reduction to a level lower than baseline by 12 months postpartum; 16- to 18-year olds reported small decreases over time; and 19- to 21-year olds reported reductions in discrimination across pregnancy but then increases in the postpartum period. These patterns confirm the need to consider age-related change across the peripartum period, yet without information about discrimination experiences that *pre-date* pregnancy, it is not possible to disentangle age-related, from pregnancy-related change. Gaining insight into the extent to which discrimination experiences change as a function of *both* age *and* childbearing status among young Black women is important for informing the type and timing of interventions aimed at reducing postpartum depression risk. Furthermore, understanding whether experiences of discrimination *prior to* pregnancy impact maternal health outcomes is essential for framing the window within which to assess potential threats to postpartum health.

In this study, we examine associations between interpersonal discrimination experienced before, during, and after pregnancy and postpartum depression in a sample of primiparous Black women. Informed by existing literature, we hypothesized that experiences of discrimination would increase across the transition to parenthood (spanning a period from 2 years pre-pregnancy to 1-year postpartum). In addition, we expected changes to be age-related, such that younger women would experience greater increases in discrimination over time. We then used a rigorous pairwise matching approach to test the hypothesis that increasing experiences of discrimination would be observed among childbearing women, but not among non-pregnant women, assessed across the same age interval. Finally, we hypothesized that greater increases in discrimination experiences would predict more severe depressive symptoms, particularly for the childbearing group.

## Method

### Participants

The sample included participants from the population-based Pittsburgh Girls Study (PGS), an accelerated longitudinal study of 2,450 women recruited as children into four age cohorts (5, 6, 7, and 8 years old) between 1999 and 2000. The PGS sample was formed following a city-wide enumeration of 103,238 households in Pittsburgh, PA. Neighborhoods in which at least 25% of the families were living at or below the poverty level were fully enumerated (i.e. all homes were contacted to determine if the household contained an eligible girl), and a random selection of households in all other city neighborhoods were 50% enumerated (Keenan et al., 2010). More than half of PGS participants (57.5%, *N* = 1,408) identified as Black (Black *n* = 1,292 and Black and other race(s) *n* = 116). Annual PGS interviews have been conducted for over 20 years, and pregnancies and birth outcomes have been tracked since early adolescence. Across the duration of the study, participant retention has been high: 87.4% of the original sample was retained at the close of annual assessment wave 20.

The present analysis focused on a PGS sub-sample of 670 participants who identified as Black (1.8% Hispanic): 335 were first-time mothers, and 335 were non-pregnant matched controls (*s*ee details below in *Pairwise Matching*). Participants contributed data from four PGS assessment waves, defined using anchors for the childbearing individuals: 2 years prior to their pregnancy (T − 2), 1 year prior to pregnancy (T − 1), during pregnancy (T), and 1-year postpartum (T + 1). The T − 1 timepoint reflected the PGS assessment that occurred before the delivery date minus 40 weeks; T − 2 was the PGS assessment 1 year prior. Data used in the current analysis were collected between 2010 and 2020.

### Procedure

Approval for all study procedures was obtained from the University of Pittsburgh Institutional Review Board. Written informed consent from the caregiver and verbal assent from the girl were obtained through age 17. Starting at age 18, all participants provided written informed consent. Annual in-home interviews were conducted (separately for the child and caregiver when girls were < 18 years) by trained field interviewers using laptop computers (Keenan et al., 2010). The interviewers were trained to be professional, respectful, and empathic. Participants were financially reimbursed for their help with the research.

### Measures

*Experiences of discrimination.* Participants completed the Everyday Discrimination Scale (EDS; Williams, Yu, Jackson, & Anderson, 1997) annually after the measure were introduced into the PGS protocol in 2010 (participant ages 15–18 years). The EDS is a 9-item scale that assesses the frequency of various forms of interpersonal mistreatment in daily life (e.g. ‘You were treated with less respect than other people’ ‘People acted as if they thought you were dishonest’). Items were scored on 4-point Likert scales (1 = never to 4 = often; range = 9–36) and summed to indicate frequency of experienced discrimination. The EDS had good internal consistency, with Cronbach’s alpha ranging from 0.87 (T − 2) to 0.90 (T).

*Depressive symptoms.* Self-reported depressive symptoms at T − 2 and T + 1 were measured using the Adolescent Symptom Inventory-4 (ASI-4; Gadow & Sprafkin, 1998) administered through 17 years and transitioning to the Adult Self-Report Inventory (ASRI-4; Gadow, Sprafkin, & Weiss, 2004) at age 18. Participants rated the frequency of all nine DSM-IV symptoms of major depressive disorder plus feelings of hopelessness on 4-point scales (0 = never to 3 = very often). The 10 items were summed to form a depression severity score (range = 0–30). The ASI-4/ASRI-4 depression scale demonstrates convergent and discriminant validity and differentiates between clinical and non-clinical samples (Gadow et al., 2004). T-scores >60 denote clinically significant depression severity. Internal consistency was 0.89 at T − 2 and 0.87 at T + 1.

*Covariates.* Date of delivery was reported by participants and obtained from electronic medical records (available for 62% of the sample). Maternal age at delivery was calculated from the date of delivery relative to the date of birth. Participants reported on receipt of public assistance (e.g. Supplemental Nutrition Assistance Program, Temporary Assistance for Needy Families). Life stress (e.g. financial problems, inadequate housing, exposure to violence) was assessed using the 28-item Difficult Life Circumstances scale (Barnard, 1994) at time T − 2. Items were each scored as 0 = No or 1 = Yes and summed to generate a total score. Participants reported on weekly use of alcohol, tobacco, and other substances (e.g. marijuana, opioids, sedative, and stimulants) using the Nicotine Alcohol and Drug Use measure (Pandina, Labouvie, & White, 1984) administered at time T − 2.

### Data analytic plan

*Pairwise matching.* An algorithm was created in RStudio (RStudio Team, 2020) to match childbearing participants pairwise with non-pregnant participants on (1) racial identity, (2) PGS cohort, (3) age in years, and (4) EDS total score at the PGS wave corresponding to 2 years prior to pregnancy (T − 2). When exact matches could not be found, the algorithm allowed a series of steps to vary the EDS score; first seeking a match in non-pregnant participants with EDS scores one point higher (+1), then one point lower (−1), then two points higher (+2) and then two points lower (−2). If a match could still not be found after completing these steps, the matching process was stopped. Race, PGS cohort, and age in years were not allowed to vary as matching criteria. Matched controls had not become pregnant during the specified period from T − 2 to T + 1. The random matching algorithm was run 10,000 times to identify pairwise matches with the maximum numbers of matching cases.

By January 2020, 822 first-time mothers had been identified in the PGS sample, of whom 604 identified as Black or Black and another race. Of this sample, 237 participants were excluded because the T − 2 timepoint pre-dated the introduction of the EDS measure into the PGS protocol. Of the 367 eligible childbearing participants, 13 (3.5%) were excluded because the timing of the pregnancy relative to a PGS assessment could not be verified. Furthermore, to be included in the present analysis, participants needed to contribute EDS data at T − 2 and one other timepoint. Thus, an additional nine participants (2.5%) missing all follow-up data were excluded. Among the remaining 345 primiparous participants, exact non-pregnant matches were identified for *N* = 296 (85.8%). An additional 19 were matched on an EDS score of +1 (5.5%), 13 were matched at −1 (3.8%), 5 were matched at +2 (1.4%), and 2 were matched at −2 (0.1%). No match could be identified for 10 participants (3.4%). Thus, the final analytic sample included *N* = 670 matched pairs (335 childbearing participants and 335 non-pregnant controls).

*Statistical analyses.* Childbearing versus non-pregnant groups were compared on sociodemographic and study variables using ANOVAs/t-tests and chi-square analyses in SPSS version 28. Life stress and use of alcohol, tobacco, and other substances were each associated with the intercept (T − 2 discrimination) and/or the dependent variable (T + 1 depression) and so were included as covariates in subsequent analyses.

Linear growth curve (LGC) analysis was conducted using Mplus version 8.8 to examine trajectories of discrimination experiences from 2 years prior to pregnancy (T − 2; intercept) to 1-year postpartum (T + 1) in childbearing participants (*N* = 335). This unconditional LGC model with four repeated measurements of discrimination assumed that no other predictors accounted for the variation of intercept and slope (e.g. Curran, 2000). Model fit was evaluated using conventional thresholds for the comparative fit index (CFI), the Tucker-Lewis index (TLI), the standardized root mean squared residual (SRMR), and the root-mean-square of approximation (RMSEA) (Hu & Bentler, 1999; McDonald & Ho, 2002). Participant age at T − 2 was added to the model to test the effects of age on changes in discrimination across the transition to parenthood. Mplus conducts multiple imputation of missing data using Bayesian estimation methods (Asparouhov & Muthén, 2010).

Using a matched pair design, we first conducted unconditional growth mixture modeling (GMM) to examine whether discrimination experiences changed as a function of pregnancy by comparing the reports of childbearing and non-pregnant individuals across the same interval (from T − 2 through T + 1). A Wald test of parameter constraints was conducted to assess for group differences in the slope. Depressive symptom severity at T + 1 was then added to the growth mixture model as a continuous outcome variable to test the effects of T − 2 discrimination (intercept) and changes in experiences of discrimination from T − 2 through T + 1 (slope) on depression severity. In the final steps, we adjusted for pre-pregnancy (T − 2) depressive symptom severity and also included T − 2 covariates.

## Results

### Descriptive information

In the sample of 335 first-time mothers, 270 (80.6%) contributed data across all four time points from pre-pregnancy to postpartum, and 41 (12.2%) and 24 (7.2%) contributed data at three, and at two, time points, respectively. Among the non-pregnant participants, 257 (76.7%) contributed data across all time points, and 42 (12.5%) and 36 (10.8%) contributed data at three, and at two, time points, respectively.

Descriptive statistics are presented in Table 1. The mean age of the childbearing participants at the time of delivery was 22.32 years (SD = 2.71, range = 17–30), and the mean timing of the postpartum (T + 1) assessment was at 13.6 months (SD = 3.45, range = 7.5–27.1). By design, there were no differences between the childbearing and non-pregnant groups on racial identity, PGS cohort, age, or EDS score at time T − 2. There were also no differences between groups in potentially confounding psychosocial factors (receipt of public assistance, life stress, and depressive symptom severity) at baseline. However, childbearing participants were more likely than their non-pregnant peers to report substance use at time T − 2. In comparison to the childbearing group, the non-pregnant matched controls reported more experiences of discrimination at T (*t* = 2.64, *p* < .01) and T + 1 (*t* = 3.09, *p* < .01), and greater depression severity at T + 1 (*t* = 2.17, *p* < .05). At T + 1, 28% of the childbearing and 21% of the non-pregnant group reported depression severity within the clinical range.

**Table 1.** Descriptive statistics for childbearing and non-pregnant participants

	Childbearing group		Non-pregnant group			
	*N* (%)	Mean (SD)	*N* (%)	Mean (SD)	t-test/X^2^	
Race: Black*^a^*	319 (95.2)		319 (95.2)			
Black and another race	16 (4.8)		16 (4.8)		.00	
Ethnicity: Hispanic	6 (1.8)		6 (1.8)		.00	
Public assistance at T − 2	11 (4.23)		16 (6.02)		.86	
Age at T − 2 (years)*^a^*		19.92 (2.62)		20.08 (2.76)	.74	
Discrimination at T − 2*^a^*		14.0 (4.74)		14.0 (4.71)	.10	
Depression severity at T − 2		6.67 (4.77)		6.52 (4.49)	− .42	
Life stress at T − 2		2.21 (2.18)		2.16 (2.27)	− .29	
Alcohol use at T − 2	60 (17.91)		37 (11.82)		4.71*	
Tobacco use at T − 2	53 (15.82)		15 (4.80)		20.95**	
Other substance use at T − 2	73 (21.79)		41 (13.10)		31.20**	
Discrimination at T − 1		14.30 (5.14)		14.38 (4.95)	.20	
Discrimination at T		13.70 (4.82)		14.76 (5.00)	2.64**	
Age at delivery (years)		22.32 (2.71)		–	–	
Postpartum months at T + 1		13.63 (3.45)		–	–	
Discrimination at T + 1		13.95 (4.81)		15.26 (5.10)	3.09**	
Depression severity at T + 1		6.07 (4.39)		6.90 (5.02)	2.17*	
T − 2 to T + 1 interval (months)		38.03 (4.71)		38.13 (5.23)	.24	

*Note. ^a^*Pair-wise matching variable. Data were collected at four time points (T − 2, T − 1, T and T + 1) for both childbearing and matched non-pregnant participants. For the childbearing group, timepoints represented: 2 years prior to pregnancy (T − 2), 1 year prior to pregnancy (T − 1), pregnancy (T), and 1-year postpartum (T + 1). * p < .05; ** *p* < .01.

The analytic sample was representative of Black women in the population-based PGS in terms of discrimination experiences assessed at mean age 19 years. Thus, there were no differences in EDS score for the analytic sample (*n* = 670, mean = 14.02, SD = 4.72) compared with the remaining Black PGS participants (*n* = 615, mean = 13.93, SD = 4.65, *t* = 0.39, *ns*). As expected, childbearing participants excluded because their pre-pregnancy assessment predated the introduction of the EDS in the PGS protocol were younger at the time of delivery (mean = 17.9 years, SD = 2.19 versus mean = 22.3 years, SD = 2.71; *t* = 20.6, *p* < .001). In addition, excluded participants with available data (*n* = 179) reported higher levels of T − 2 depressive symptoms than included participants (mean = 7.92, SD = 4.58 versus mean = 6.69, SD = 4.76; *t* = 2.85, *p* < .01). An additional 19 participants (i.e. missing follow-up data or could not be matched) were older than the included participants (Cohen’s *d* = 0.57) but otherwise did not differ on any study variables.

### Changes in discrimination experiences across the transition to parenthood

The unconditional LGC model fit the data well (CFI = .978, TLI = .974, SRMR = .043, RMSEA = .079; Table 2). As shown in Table 3, the LGC model had a significant mean intercept (*B_I_* = 14.1, *SE* = .253, *p* < .001) but a non-significant estimated slope (*B_S_* = .038, *SE* = .090, *ns*) indicating no overall change in discrimination experiences across the transition to parenthood. However, there was substantial between-person variability in intercept (*D_I_* = 15.8, *SE* = 1.78, *p* < .001) and slope (*D_S_* = .760, *SE* = .300, *p* < .05). When age at T − 2 was included as a predictor in the LGC model, results showed that older age was associated with more experiences of discrimination at baseline (*B_I_* = .646, *SE* = .091, *p* < .001); however, younger age at baseline was associated with more rapidly increasing experiences of discrimination across the transition to parenthood (*B_S_* = − .142, *SE* = .037, *p* < .001).

**Table 2.** Model fit indices of LGCMs and GMMs among childbearing and non-pregnant groups

Covariates		Model fit indices		
		RMSEA	CFI/TLI	SRMR
LGCM: Childbearing group				
None (unconditional model)		.079	.978/.974	.043
Age		.061	.983/.976	.037
		Log likelihood	AIC	BIC
GMM: childbearing and non-pregnant groups	Outcome			
None (unconditional model)	None	− 7423.5	14871.0	14925.1
None (unconditional model)	Depression at T + 1	− 9115.5	18268.9	18354.5
Depression severity at T − 2	Depression at T + 1	− 8727.9	17495.8	17585.3
Depression severity at T − 2, Life stress, alcohol, tobacco and other substance use at T − 2	Depression at T + 1	− 8013.8	16083.5	16206.6

**Table 3.** Results of LGCMs and GMMs predicting depression at T + 1 among childbearing and non-pregnant groups

Covariates		Model results						
		*B*_*I*_ *(SE)*	*p*	*95% CI*	*B_S_ (SE)*	*p*	*95% CI*	*Wald X^2^ (p)*
LGCM: childbearing group								
None (unconditional model)		14.1 (.253)	.000	13.6–14.5	.038 (.090)	.671	− .110–.187	–
Age		.646 (.091)	.000	.497–.795	− .142 (.037)	.000	− .202–.081	–
GMM: childbearing group	Outcome							
None (unconditional model)	None	14.1 (.253)	.000	13.6–14.5	.044 (.111)	.690	− .114–.187	12.0 (<.001)
None (unconditional model)	Depression at T + 1	.630 (.075)	.000	.506–.754	2.37 (.502)	.000	1.54–3.19	–
Depression severity at T − 2	Depression at T + 1	.626 (.077)	.000	.500–.753	2.55 (.939)	.007	1.01–4.10	–
Depression severity at T − 2, Life stress, alcohol, tobacco and other substance use at T − 2	Depression at T + 1	.621 (.081)	.000	.487–.754	2.34 (.803)	.004	1.02–3.66	
GMM: non-pregnant group	Outcome							
None (unconditional model)	None	14.0 (.252)	.000	13.6–14.4	.480 (.090)	.000	.332–.628	12.0 (<.001)
None (unconditional model)	Depression at T + 1	.815 (.071)	.000	.698–.933	1.59 (1.11)	.153	− .239–3.412	–
Depression severity at T − 2	Depression at T + 1	.912 (.081)	.000	.779–1.05	− .130 (1.23)	.916	− 2.15–1.89	–
Depression severity at T − 2, life stress, alcohol, tobacco and other substance use at T − 2	Depression at T + 1	.914 (.095)	.000	.758–1.07	− .029 (1.21)	.981	− 2.03–1.97	

### Changes in discrimination experiences as a function of childbearing

We used a growth mixture model to examine changes in discrimination experiences as a function of childbearing. Results of the Wald test showed a significant difference in discrimination experiences over time by group (*X^2^*_slope_ = 12.0, *df* = 1, *p* < .001). In contrast to the non-significant change in discrimination among childbearing participants, discrimination experiences increased linearly with age across the same developmental period among the non-pregnant participants (*B_S_* = .480, *SE* = .090, *p* < .001; Table 3). The non-pregnant group also had a significant mean intercept (*B_I_* = 14.0, *SE* = .252, *p* < .001).

### Association between changes in discrimination experiences and depression severity

Depressive symptom severity at T + 1 was added to the growth mixture model to examine whether changes in discrimination were associated with later depressive symptomatology. Results are summarized in Table 3. For the childbearing group, greater discrimination experiences at T − 2 (intercept) and increasing experiences of discrimination across the transition to parenthood (T − 2 to T + 1; slope) predicted greater postpartum depressive symptom severity (T + 1; *B_I_* = .630, *SE* = .075, *p* < .001; *B_S_* = 2.37, *SE* = .502, *p* < .001). In the non-pregnant group, greater discrimination at T − 2 predicted depressive symptom severity at T + 1 (*B_I_* = .815, *SE* = .071, *p* < .001), but increasing discrimination from T − 2 to T + 1 was unrelated to depression severity at T + 1 (*B_S_* = 1.59, *SE* = 1.11, *p* = .153).

Next, we accounted for within-person stability in depression by covarying depression severity at T − 2. Results for both groups were unchanged: for the childbearing participants, greater discrimination experiences at baseline (T − 2; intercept) and increasing experiences of discrimination across the transition to parenthood (T − 2 to T + 1; slope) predicted greater postpartum depressive symptom severity (T + 1; *B_I_* = .626, SE = .077, *p* < .001; *B_S_* = 2.55, SE = .939, *p* < .01). Akaike information criterion (AIC) and Bayesian information criterion (BIC) model fit criteria improved when T − 2 depression severity was added to the model (Table 2). Among non-pregnant participants, greater discrimination at T − 2 (intercept) predicted depressive symptom severity at T + 1 (*B_I_* = .912, SE = .081, *p* < .001), but changes in discrimination experiences from T − 2 to T + 1 were not associated with depression severity at T + 1 (*B_S_* = − .130, SE = 1.23, *p* = .916).

**Figure 1.**
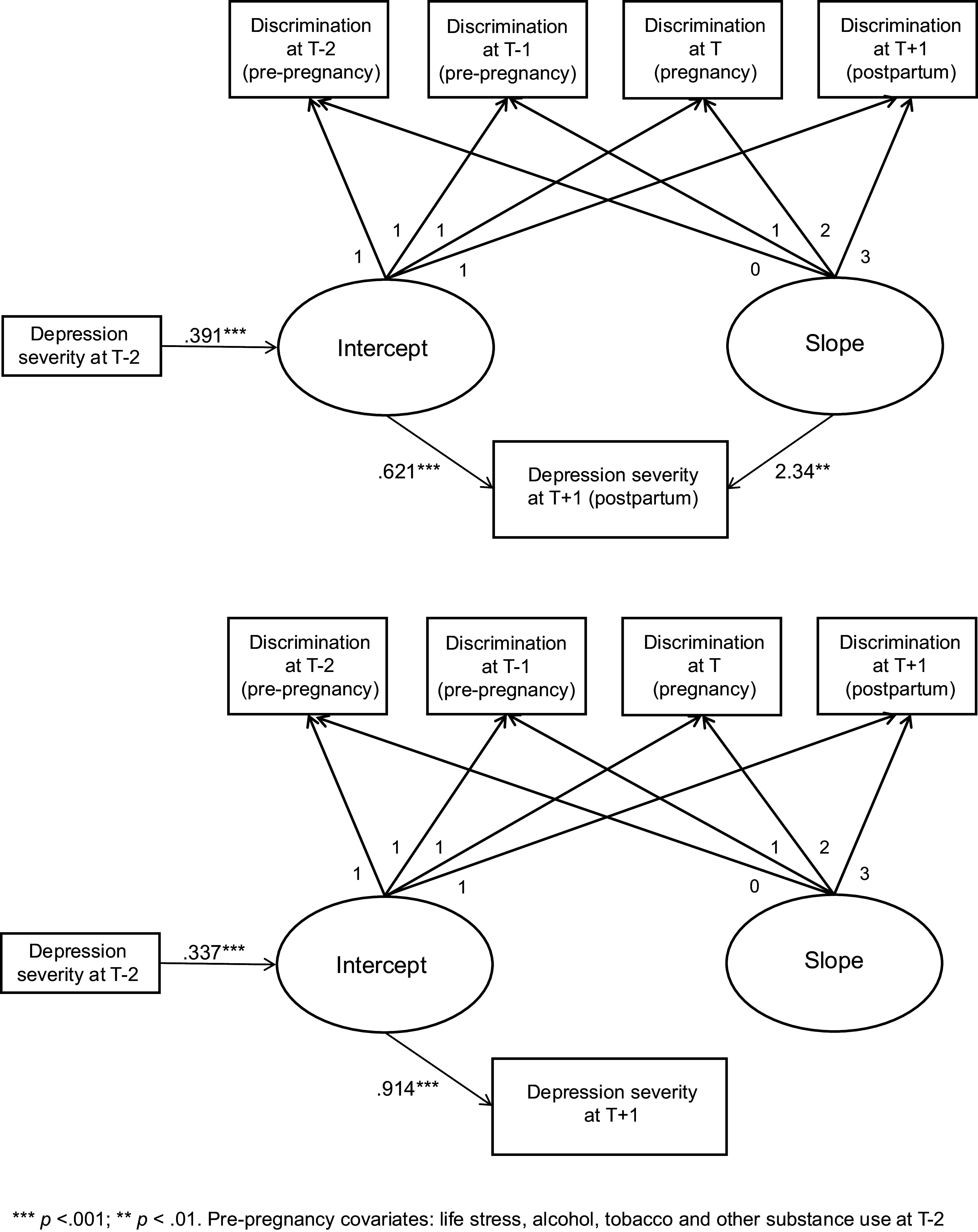
Final conditional latent growth curve models with *T* + 1 depression severity as a continuous outcome for childbearing individuals (top) and non-pregnant matched controls (bottom).

In the final step, we included T − 2 life stress, alcohol, tobacco, and other substance use as covariates. Model fit improved, but the results were unchanged. For the childbearing participants, greater discrimination experiences at baseline (T − 2; intercept) and increasing experiences of discrimination across the transition to parenthood (T − 2 to T + 1; slope) predicted higher levels of postpartum depressive symptom severity (T + 1; *B_I_* = .616, SE = .079, *p* < .001; *B_S_* = 2.29, SE = .740, *p* < .01), as shown in Table 3. Among non-pregnant participants, greater discrimination at T − 2 (intercept) predicted depressive symptom severity at T + 1 (*B_I_* = .905, SE = .095, *p* < .001), but T − 2 to T + 1 changes in discrimination experiences were unrelated to depressive symptom severity at T + 1 (*B_S_* = − .194, SE = 1.20, *p* = .871). The final conditional LGC models are depicted in Figure 1.

## Discussion

This study showed that primiparous Black women who experience increasing discrimination across the transition to parenthood reported higher levels of postpartum depressive symptoms. Key strengths of the study included the availability of repeated-measures data on discrimination experiences that fully spanned the period from pre-pregnancy through the first postpartum year in a population-based cohort with high participant retention. In addition, the large PGS sample provided a unique opportunity for pairwise matching of individuals who did not become pregnant across the same interval to allow examination of changes in discrimination as a function of *both* age *and* childbearing status.

Four main findings emerged. First, there was substantial heterogeneity in the experiences of discrimination among childbearing individuals before pregnancy and across time but no overall increase in discrimination across the transition to pregnancy at the group level. While not supporting our hypothesis, this finding aligns with prior work following a young, racially diverse pregnant sample through 12 months postpartum that also reported large individual differences in trajectories of discrimination (Rosenthal et al., 2015). This extends these results to show that, in a sample of Black first-time mothers, experiences of discrimination are also highly variable across the preconception, prenatal, and postpartum periods.

Second, our findings revealed that changes in discrimination experiences are age-related. Younger Black childbearing women reported steeper increases in discrimination over time, a finding shown previously with non-childbearing adolescent and young adult populations (e.g. Greene et al., 2006; Seaton et al., 2008; Williams & Mohammed, 2009). There may be several explanations for this developmental pattern. For example, negative societal stereotypes regarding sexuality and Black womanhood may be most apparent in early adulthood (Collins, 2000), a critical period of identity development. Heightened awareness of subtle forms of bias also develops in late adolescence (Seaton, Yip, & Sellers, 2009). In addition, emerging adulthood typically involves expanded social roles and increased engagement with broader societal institutions, potentially leading to greater exposure to discriminatory experiences (Arnett, 2000; Hope, Hoggard, & Thomas, 2015). Younger women may also be more attuned to changes in discriminatory encounters as they navigate new social contexts (Sellers & Shelton, 2003).

Third, study findings showed that experiences of discrimination vary as a function of childbearing after accounting for age, maturation, and baseline levels of discrimination but not in the direction expected. Thus, relative to the *increasing* trajectory observed among matched, non-pregnant participants across the same time interval, the unchanged slope of discrimination experiences among the childbearing group suggests that pregnancy and the first-year postpartum could represent a period of relative reprieve. One possibility is that supportive infrastructures become more available in the transition to parenthood. Support may be manifest in improved family relations and extended caregiving networks, enhanced friendships, and access to supportive health personnel (e.g. doulas) that help limit exposure and/or strengthen resilience to discrimination. It is also possible that major social, educational, and occupational transitions in early adulthood contributed to group differences in geographic mobility and economic opportunities that led to divergent discrimination experiences. Given that Pittsburgh is a largely segregated city (Pittsburgh Neighborhood Project, 2019), greater mobility across racially mixed neighborhoods among individuals in the non-pregnant group could have exposed them to more frequent interactions with non-Black individuals and a greater likelihood of experiencing discrimination (Dailey, Kasl, Holford, Lewis, & Jones, 2010; Hunt, Wise, Jipguep, Cozier, & Rosenberg, 2007). Given the wide age range of participants included in the current study, comparable data on supportive infrastructure and occupational experiences were not uniformly available in the PGS. However, investigation of these factors as mediators and effect modifiers will be important for future work.

Fourth, results showed that both pre-pregnancy and increasing experiences of discrimination across the transition to parenthood predicted more severe postpartum depressive symptoms. These prospective associations extend findings from cross-sectional studies with Black pregnant and postpartum women (Heldreth et al., 2016; Weeks et al., 2022) by revealing that discrimination experiences *prior to* pregnancy have the potential to impact postpartum health. Furthermore, our findings suggest that even relatively low levels of interpersonal discrimination can make a difference for postpartum depression risk. Importantly, our analyses showed that increasing experiences of discrimination were unrelated to later depression among non-pregnant individuals. These differential results were not explained by group differences in pre-existing depression and were unchanged when within-person stability and additional covariates were accounted for. This raises the possibility of pregnancy-specific mechanistic pathways. For example, given that pregnancy is recognized as a ‘natural physiological stress test’ (Rich-Edwards, Fraser, Lawlor, & Catov, 2014), increasing exposure to discrimination in the context of childbearing may have particularly insidious effects on physiological wear-and-tear and alterations in stress response systems that undermine health. Large-scale cohort studies that begin prior to pregnancy are needed to test this hypothesis.

Our findings also need to be considered in the context of some limitations. First, the EDS measure did not distinguish the source of discrimination, so our results may reflect the unique intersectional experiences of Black women rather than experiences specific to racial discrimination. Prior work has also shown that young, urban women of color experience discrimination associated with their age (Kimmel, 1988), a finding that could account for the age-related associations reported here. Second, we focused on trajectories of interpersonal discrimination in this study, but we recognize that it is the accumulation of multiple interacting risk and promotive factors that impact mental and physical health (Geronimus et al., 2006). Some of those factors were not measured in all participants (e.g. emotional/instrumental support from peers), and some were not included in the PGS protocol (e.g. workplace characteristics, access to doula care). Much work is needed to understand the dynamic interplay of specific stress exposures to identify targets that could buffer and support postpartum health. Third, the current results reflect the experiences of primiparous Black mothers living in a single urban region and a largely segregated city, which may limit generalizability. The analyses also excluded participants who were younger at delivery and did not have the opportunity to report on discrimination experiences. These individuals reported more depressive symptoms prior to pregnancy and may have differed in other important ways that could have impacted the associations reported. The small number of other excluded participants (e.g. missing follow-up data, no identified match) likely had minimal impact on sample representativeness. Nevertheless, future work should explore the consistency of results for multiparous women and across diverse populations with respect to age, differences in racial and ethnic identity, and geography. Finally, measurement continuity is a considerable challenge for understanding peripartum depression within a lifespan context. In this study, we prioritized repeated measurement with a DSM-based checklist to parallel assessment in clinical settings and allow for direct comparisons with non-pregnant individuals. However, this approach may have confounded symptoms associated with the postpartum state (e.g. loss of sleep, changes in weight). In addition, there was variability in the timing of the postpartum (T + 1) assessments that ranged from 7 to 27 months following delivery. Although postpartum depression is generally defined as occurring within the first year (O’Hara & McCabe, 2013), the extended window allowed capture of depressive symptoms that often persist or worsen beyond 2 years postpartum (Baron, Bass, Murray, Schneider, & Lund, 2017; Putnick et al., 2020).

## Conclusions

Understanding relationships between discrimination experiences and postpartum depression is critical for developing interventions, programs, and policies to reduce racial disparities in maternal mental health. The current results suggest that the transition to parenthood may be a life stage when young Black women experience relatively less discrimination. Yet individuals who experience high levels of discrimination pre-pregnancy and experiences that increase across the peripartum period may be especially vulnerable to postpartum depression. Finding solutions to individual, structural, and systemic discrimination impacting women before, during, and after pregnancy is challenging, yet evidence highlights promising approaches such as enhancement of resilience factors with culturally sensitive social support interventions that foster positive racial identity (Hurd, 2024). Research with pregnant women has also shown that talking to others about unfair treatment may lower the risk of depression during pregnancy (Ertel et al., 2012), and informal sources of support (e.g. spouse, family, and friends) may be especially valuable for women who have limited access to health resources and opportunities (Himmelstein & Himmelstein, 2020; McLeish & Redshaw, 2017; Phelan & Link, 2015). Finally, reproductive justice frameworks such as the Black Mamas Matter Alliance (Muse, 2018) play a critical role in advocating for maternal care knowledge to support autonomy among marginalized patients and for investment in paraprofessionals such as health visitors and doulas to reduce the negative effects of discrimination on the health of both mother and child.
